# Effects of DAPAgliflozin on CARDiac substrate uptake, myocardial efficiency, and myocardial contractile work in type 2 diabetes patients—a description of the DAPACARD study

**DOI:** 10.1080/03009734.2018.1515281

**Published:** 2019-01-08

**Authors:** Axel Åkerblom, Jonas Oldgren, Aino Latva-Rasku, Lars Johansson, Vera Lisovskaja, Cecilia Karlsson, Jan Oscarsson, Pirjo Nuutila

**Affiliations:** a Uppsala Clinical Research Center and Department of Medical Sciences, Uppsala University, Uppsala, Sweden;; b Turku PET Centre, University of Turku, Turku, Finland;; c Department of endocrinology, Turku University Hospital, Turku, Finland;; d Antaros Medical AB, Gothenburg, Sweden;; e AstraZeneca, Gothenburg, Sweden

**Keywords:** Diabetes, experimental diabetes, magnetic resonance, molecular biology, nuclear medicine

## Abstract

**Background:** Diabetes increases the risk for cardiovascular (CV) events. It has recently been shown that the use of sodium-glucose cotransporter 2 (SGLT2) inhibitors leads to a reduction in CV outcomes in patients with type 2 diabetes mellitus (T2DM), including mortality and heart failure hospitalization. The exact mechanisms of how SGLT2 inhibitors lead to this CV risk reduction remain incompletely understood. The study of DAPAgliflozin on CARDiac substrate uptake, myocardial efficiency and myocardial contractile work in type 2 diabetes patients (DAPACARD) (NCT03387683) explores the possible effects of dapagliflozin, an SGLT2 inhibitor, on cardiac work, metabolism, and biomarker levels.

**Methods:** DAPACARD is an international, randomized, double-blind trial that aims to examine the effects of dapagliflozin versus matching placebo in 52 patients with T2DM that are on stable metformin therapy prior to and during the 6 weeks of treatment. The primary efficacy endpoint is change in global longitudinal strain of the left ventricle (GLSLV) measured with magnetic resonance imaging (MRI) between baseline (pre-treatment) and end of study (on-treatment). The secondary endpoint is the corresponding change in myocardial efficiency measured as external left ventricular work divided by total left ventricular work, which is estimated using [11C]-acetate clearance using positron emission tomography (PET).

**Conclusion:** The DAPACARD study is an extensive investigation of cardiac function and metabolism, by advanced imaging with PET and MRI, as well as biomarkers, performed in order to further explore how the SGLT2 inhibitor dapagliflozin could influence cardiovascular outcomes in patients with T2DM.

## Background

Type 2 diabetes mellitus (T2DM) is associated with a 2–4-fold increased risk for cardiovascular (CV) events as compared to patients without T2DM (1). Although antidiabetic drugs reduce hyperglycemic complications and morbidity, to date few antidiabetic drugs have been proven to reduce CV mortality (2–4). The sodium-glucose cotransporter 2 (SGLT2) inhibitor empagliflozin showed a reduction in CV outcomes, including CV mortality and heart failure admissions, in patients with T2DM (4). Another SGLT2 inhibitor, canagliflozin, recently showed similar results including a decrease in heart failure admissions, but not in all-cause mortality, in patients with T2DM (5,6). For both of the SGLT2 inhibitors, the positive cardiovascular effects became apparent within months from treatment start, suggesting that mechanisms beyond improved glucose control and reduced atherosclerosis are involved in the CV risk reduction (5). The effect of dapagliflozin on CV outcomes in a broad T2DM population is currently investigated in a large phase III study (Multicenter Trial to Evaluate the Effect of Dapagliflozin on the Incidence of Cardiovascular Events—DECLARE-TIMI 58 [NCT01730534]), with results expected in the second half of 2018 (7).

A number of potential mechanisms have been suggested to explain the CV benefits observed in studies with SGLT2 inhibitors. The primary effect of these drugs is a reduction in reabsorption of glucose in proximal tubuli, which results in increased urinary excretion of glucose and sodium and thus increased diuresis. Consequently, SGLT2 inhibition not only leads to a decrease in hemoglobin A1c (HbA1c) levels, but also a lower body weight and lower blood pressure, as well as an increase in hematocrit (Hct) levels (4,5,8). However, none of these effects is believed to fully explain the reported CV benefits. The need for mechanistic studies is therefore warranted to elucidate the mechanisms behind the beneficial effects of SGLT2 inhibitors on CV outcomes (8).

Global longitudinal strain of the left ventricle of the heart (GLSLV) is a marker of cardiomyocyte contractile work of the heart. A decrease in GLSLV has been associated with CV events, including heart failure and a composite of CV death, myocardial infarction, or stroke (9).

Changes in myocardial efficiency and contractile work can be measured with positron emission tomography (PET) and magnetic resonance imaging (MRI), respectively (10–12). It has previously been shown that patients with T2DM have a reduced myocardial efficiency both in the fasting state and during insulin infusion (10,13).

In the current ‘Effects of DAPAgliflozin on CARDiac substrate uptake, myocardial efficiency and myocardial contractile work in type 2 diabetes patients—a description of the DAPACARD study’ (NCT03387683) we aim to further explore the effects of dapagliflozin on myocardial metabolism and contractility with advanced PET and MRI scans, as well as with an extensive array of biomarkers.

## The DAPACARD study

### Study objectives

The objective of the study is to explore the potential effects of dapagliflozin on myocardial perfusion, substrate uptake, and measures of contractile performance of the myocardium including left ventricle (LV) strain, as a surrogate for myofibril contractile work.

### Study design

The DAPACARD is an international, randomized, double-blind, parallel-group, phase IV trial that aims to include 52 patients with T2DM on a stable dose of metformin, randomized to 6 weeks of treatment with either dapagliflozin 10 mg once daily (OD) or matching placebo.

**Figure 1.**
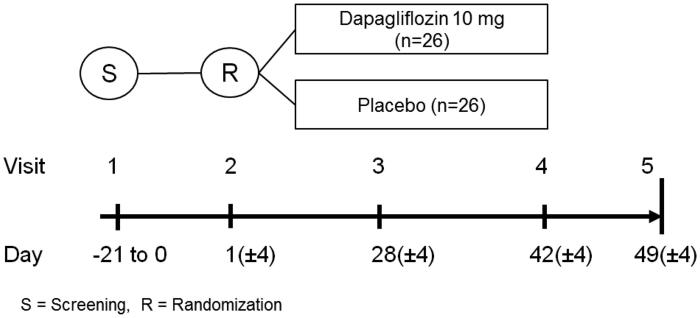
Overview of the DAPACARD trial. Screening visit (visit 1) is 0 to 21 days prior to randomization. The randomization (visit 2) is day 1, and the patients perform both PET and MRI scans prior to starting the randomized treatment. Visit 3 is a telephone call. Visit 4 is with both PET and MRI scans. A final follow-up call (visit 5) is also planned.

The study design is depicted in Figure 1. For each patient, five visits are planned. The screening visit (visit 1) takes place 0–21 days prior to randomization and start of treatment (visit 2), and is primarily aimed at determining the suitability of an inclusion of a patient in the study and collection of background information. The efficacy data will be collected at the randomization (visit 2; baseline) and end of treatment visit (visit 4, which takes place after 6 weeks on study drug). During these two visits blood and urine samples for biomarkers are collected, and PET and MRI scans are performed. It is required that patients should be fasting prior to collection of these measurements. PET and MRI scans can, if preferred, be performed on two consecutive days. A cross-over study design was discussed; however, this would have prolonged the study period for participants with a risk for an increased number of drop-outs and would have exposed the participants to additional PET scans (higher radiation exposure), and was consequently discarded.

Finally, there are two safety check-up visits planned: visit 3 (28 days after the start of the randomized treatment) and visit 5 (1 week after the cessation of the study drug). Both of these visits are scheduled telephone calls.

A steering committee with academic experts and representatives from the sponsor (AstraZeneca) and from Antaros Medical (Gothenburg, Sweden) designed the DAPACARD study in collaboration. The steering committee, together with project managers at Uppsala Clinical Research Center (Uppsala, Sweden), oversees the medical, scientific, and operational conduct of the study. The steering committee, together with statisticians, has planned the analyses and will perform the final statistical analyses.

The study adheres to the ethical principles of the Declaration of Helsinki, and to good clinical practice. The study protocol has been approved by an independent ethics committee and/or institutional review board at each site. The protocol requires investigators to obtain each subject’s signed informed consent before initiating study procedures or recording any information into the study database.

### Blood samples

A predefined set of safety blood samples will be collected at screening, randomization, and at the 6 weeks visit. Furthermore, blood samples for biomarker collection will be obtained at randomization and 6 weeks visits. The biomarkers (such as glucose, HbA1c, non-esterified fatty acids, beta-hydroxybutyrate, Hct, NT-proBNP, and FGF21) could help to understand changes in metabolism and/or function of the heart.

### Study population, study conduct, and follow-up

The study subjects are recruited either from outpatient visits to the participating centers or via registries of patients previously admitted to the participating centers.

The patients have to meet all the inclusion criteria (Table 1) before randomization, including: age of ≥40 years but <75 years, T2DM on a stable dose of metformin for at least 6 weeks, and willing to undergo both PET and MRI scans. Patients are allowed to have had coronary artery disease, but no ongoing cardiac signs or symptoms (e.g. angina pectoris, dyspnea or fatigue on exertion). No patients with decreased LVEF or overt heart failure are allowed. A complete list of the predefined exclusion criteria can be found in Table 2.

**Table 1.** Inclusion criteria.

Provision of signed and dated, written informed consent prior to any study-specific procedures, and:
1. Females or males ≥40 years up to 75 years of age.
2. Patients with type 2 diabetes on stable dose of metformin for at least 6 weeks prior to screening and HbA1c at screening visit of ≥42 mmol/mol and ≤75 mmol/mol.
3. No significant signs or symptoms of coronary artery disease or, if known coronary artery disease, currently free of symptoms^a^ and (i) all major epicardial vessels with <50% stenosis within 12 months prior to screening, or (ii) if revascularized, with all major epicardial vessels with <50% remaining stenosis after stenting or bypass surgery procedure between 3 and 12 months prior to screening.
4. Normal left ventricular ejection fraction (≥50%) assessed within 1 year prior to informed consent, and, if applicable, after most recent acute episode of coronary artery syndrome, or at screening visit.
5. Subjects with BMI ≥25 kg/m^2^.
6. Female subjects must be 1 year post-menopausal, surgically sterile, or using an acceptable method of contraception (an acceptable method of contraception is defined as a barrier method in conjunction with a spermicide) for the duration of the study (from the time they sign consent) to prevent pregnancy.^b^

^a^Cardiac symptoms include, but are not limited to, angina pectoris, dyspnea, and fatigue on exertion judged by a physician to be of cardiac origin.

^b^In addition, oral contraceptives, approved contraceptive implant, long-term injectable contraception, intrauterine device, or tubal ligation are allowed. Oral contraception alone is not acceptable; additional barrier methods in conjunction with spermicide must be used.

**Table 2.** Exclusion criteria.

Medical conditions:
1. Blood pressure at screening that would require a change in blood pressure treatment over the study period or any of the following: systolic blood pressure >160 mmHg or diastolic blood pressure >100 mmHg.
2. History of stroke or other clinically significant cerebrovascular disease.
3. Any of the following cardiovascular diseases known within 3 months prior to signing the consent at enrollment: Atrial fibrillation, or other unstable or severe arrhythmia affecting heart function; Unstable heart failure or any heart failure with NYHA class III and IV; Significant valvular disease; Significant peripheral artery disease.
4. Planned cardiac surgery or angioplasty within 3 months from enrollment.
5. Clinical diagnosis of type 1 diabetes, maturity onset diabetes of the young (MODY), secondary diabetes, or diabetes insipidus.
6. Verified body weight variability of >3 kg during the 3 months before screening (by interviews).
7. Active malignancy requiring treatment at the time of visit 1 (with the exception of successfully treated basal cell or treated squamous cell carcinoma).
8. Patients with severe hepatic impairment (Child–Pugh class C).
9. Unstable or rapidly progressing renal disease.
10. Clinically significant disease or disorder which, in the opinion of the investigator, may either put the subject at risk because of participation in the study, or influence the results or the subject’s ability to participate in the study.
Prior/concomitant therapy:
11. Ongoing treatment with other antidiabetic drugs than metformin.
12. Ongoing treatment with loop diuretics.
13. Ongoing weight loss diet (hypocaloric diet) or use of weight loss agents.
14. Contraindications to dapagliflozin therapy.
15. Ongoing treatment with systemic steroids at time of informed consent or change in dosage of thyroid hormones within 6 weeks prior to informed consent or any other uncontrolled endocrine disorder except for T2DM.
Prior/concurrent clinical study experience:
16. Previous enrollment in the present study or participation in another clinical study with an investigational product during the last 1 month prior to screening.
Diagnostic assessments:
17. Estimated glomerular filtration rate (eGFR) < 45 mL/min/1.73 m^2^, based on the MDRD study equation (www.kidney.org/content/mdrd-study-equation) (eGFR =175 × (SCr) − 1.154 × (age) − 0.203 × 0.742 [if female] × 1.212 [if black]).
18. Alcohol or drug abuse within the 3 months prior to informed consent that would interfere with trial participation or any ongoing condition leading to a decreased compliance to study procedures or study drug intake.
19. Any condition in which MRI and PET are contraindicated such as, but not limited to, having a metallic implant (such as pacemaker or cochlear implant), permanent make up, claustrophobia, or BMI ≥40 kg/m^2^.
Other exclusions:
20. Involvement in the planning and/or conduct of the study (applies to AstraZeneca, UCR, and/or Antaros staff and/or staff at the study site).
21. Plasma donation within one month of screening or any blood donation/blood loss >450 mL during the 3 months prior to screening.
22. Women who has a positive pregnancy test at enrollment or randomization, or are breastfeeding.

All patients eligible for participation in the trial must receive metformin, and be on a steady dose for at least 6 weeks before enrollment. Other antidiabetic treatments, including other SGLT2 inhibitors, insulin, glitazones, pramlinitides, DPP-IV inhibitors, GLP-1 analogues, or sulfonylurea, are not allowed. Likewise, loop diuretics are not allowed in the trial.

Overall, all pre-existing concomitant medications are asked to be kept on stable doses throughout the study from 6 weeks before screening and throughout the study, including, for example, thyroid hormones, thiazides, beta-blockers and ARBs, and ACE inhibitors (any drug affecting plasma volume and/or heart function).

Study participants are asked to not intentionally try to change body weight or to attend weight loss programs, start other medications, or change diets or current rate of physical practice during the 6 weeks of study duration.

The investigational product (IP) is dapagliflozin 10 mg once daily (OD) versus placebo (matched to IP). The dapagliflozin 10 mg once daily was chosen because it is well tolerated and is approved for long-term T2DM treatment. Study treatment compliance will be assessed by the return of all unused investigational products and empty packages. Randomization between placebo and IP will be 1:1 (Figure 1).

### Study endpoints and statistics

The primary endpoint is the change in GLSLV between the baseline and the end of treatment visits, measured with MRI. Myocardial strain is a surrogate for myocardial contractile work that is dependent on ATP production capacity, which in turn is dependent on myocardial perfusion and mitochondrial function in the myocardium (14).

The secondary endpoint is change in myocardial efficiency between the baseline and the end of treatment visits. The myocardial efficiency is calculated as the mechanical work divided by the total work, with mechanical work defined as mean arterial pressure (MAP) × stroke volume (SV) × heart rate (HR)/myocardial mass. Total work is defined as the total myocardial oxygen consumption per myocardial mass measured by [11C]-acetate PET (15). The myocardial oxygen consumption is directly proportional to the clearance of [11C]-acetate. [11C]-Acetate is immediately converted into [11C]-acetyl CoA in the cell, and after entering the citric acid cycle the label 11 C is released as [11C]-CO_2_. Also, it is assumed that the myocardial washout of [11C]-acetate is a marker of the citric cycle activity and hence mitochondrial function. [11C]-Acetate PET will also be used to estimate myocardial perfusion.

A key exploratory objective in the study is to investigate the change in uptake of free fatty acids in the myocardium by measuring [18F]-FTHA uptake with PET. [18F]-FTHA PET will also be applied to investigate the effect of dapagliflozin as compared to placebo on fatty acid uptake in other tissues, e.g. the brain and the liver. [18F]-FTHA uptake will be measured in a subset of the patients as described below.

All personnel involved with the analysis of the study will remain blinded until the study is finalized and the database is locked.

A comprehensive statistical analysis plan (SAP) has been prepared. Any subsequent amendments to the analysis will be documented, with final amendments completed prior to the unblinding of the data.

### Statistical analyses—sample size estimation

The common standard deviation in estimated change from baseline of GLSLV measured using cardiac MRI is assumed to be 2.0%, which is a conservative approximation of the results found in the literature (1.73%) (16). The effect size after 6 weeks of treatment is difficult to predict, as there are no prior short interventional studies. However, based on the meta-analysis performed (9), a change in GLS of the magnitude of 1 SD has a significant association with mortality. Baseline SD of GLSLV using cardiac MRI has been shown to be 2.25 (16) and 2.5 (17). Therefore, we judge the effect of the same magnitude as the baseline SDs, namely 2% improvement in GLSLV, to be clinically significant, and used it to power the study. Based on these assumptions, it is estimated that 17 evaluable patients/arm provides 80% power at a two-sided alpha level of 0.05 to detect a treatment difference of 2% in the change from baseline in GLSLV between dapagliflozin and placebo, assuming a common standard deviation in estimated change from baseline in GLSLV of 2.0%.

Change in myocardial efficiency from baseline to the end of treatment (%, measured as external LV work per gram [defined as MAP × SV × HR/LV mass] divided by total LV work per gram [linearly proportional to MVO2, in turn linearly proportional to [11C]-acetate clearance/LV mass]), is the secondary endpoint in the DAPACARD study. Healthy subjects had in two different studies baseline myocardial efficiency of 54.3 (*n* = 36) and 49.3 (*n* = 8) (pooled mean of 50.21) mmHg · L · g^−1^, with a standard deviation (SD) of 8.9 and 14.2 mmHg · L · g^−1^, respectively (15,18). A 25% increase in myocardial efficiency is regarded as clinically significant and translates into 12.55 mmHg · L · g^−1^ with a SD of 14.2 mmHg · L · g^−1^ between dapagliflozin and placebo treatment group. Altogether 22 evaluable subjects per group are needed to detect a treatment difference of 12.55, at a two-sided significance level of 0.05 and 80% power. Assuming a 15% non-evaluable rate, a total of 52 (26 per group) subjects will be randomized.

The [18F]-FTHA examinations are exploratory and will not be performed in all the randomized patients. Instead, a minimum 40 and a maximum of 44 of the randomized patients will be scheduled for examination for [18F]-FTHA uptake in heart, liver, kidney, and the brain. This is due to ethical reasons and aims to limit unnecessary exposure to radioactivity. Viljanen et al. (19) reported SD of myocardial fatty acid uptake to be 0.4 (before diet) and 0.2 (after diet) in a healthy population, leading to a pooled SD of 0.32. No information is available regarding the magnitude of the treatment effect in the planned study, but an effect of approximately 0.3–0.4 (approximately 10% relative change) is judged to be clinically relevant. Using this assumption, and the pooled estimate of the SD as a conservative approximation of the variability of measurement differences between baseline and end of treatment, leads to approximately 80–90% power with 40 randomized patients (at a two-sided alpha level of 0.05 and assuming 15% non-evaluable rate).

### Analysis of the efficacy endpoints

All the analyses will be based using measurements that were taken in accordance to the planned procedures. Since the main objective of the study is to search for mechanisms, the per-protocol population will be used for the analyses (e.g. if a patient uses prohibited medication during the study, this patient may be removed from the analysis). As the primary, secondary, and exploratory objectives of this study are of the same nature, namely to detect a systematic difference between the dapagliflozin and the placebo groups in measurements that can be regarded as continuous, all the endpoints will be analyzed in the same manner. An ANCOVA model will be used which, besides the treatment group indicator, includes the baseline value of the respective endpoint as a co-variate. The results will be summarized through least square means (LSM) for each of the treatment groups, obtained from the model, as well as the difference thereof, with the corresponding standard deviations, confidence intervals, and, in the case of the difference in LSM, *p* values.

### Evaluation of safety

Throughout the study, safety findings, such as serious adverse events and adverse events leading to discontinuation of treatment, will be collected. These will later be summarized, with the frequencies of different types of adverse events tabulated. The details of the events will be provided in listings. Safety will also be evaluated through safety labs, and the results will be summarized in terms of the values of the labs before and after the treatment period, as well as the differences thereof.

### Study sites and collaborators

Sites are selected on the basis of availability of PET and MRI facilities with a prerequisite of the possibility to produce the radioactive PET tracers, namely [11C]-acetate and [18F]-FTHA. The facilities must be adjacent to a clinic with the capacity of including subjects for the study, based on feasibility data. Only two sites have been identified that fulfill these requirements: Uppsala University Hospital, Uppsala, Sweden and Turku University Hospital, Turku, Finland.

The Uppsala Clinical Research Center (UCR) and Antaros Medical are responsible for site and data management, as well as imaging management (including analyses), respectively. Covance Laboratories is the central lab involved in the study.

AstraZeneca is the sponsor of the study.

### Present status

The first patient was enrolled on 28 February 2018, at the Turku University Hospital, Turku, Finland. The first patient in Sweden to be recruited is scheduled for August 2018. It is expected that all the patients planned to participate in the study will be enrolled within a year of study start.

## Discussion

The beneficial effects of the SGLT2 inhibitors on CV outcomes, including CV mortality and decreased rate of heart failure admissions, is a major progress in the treatment of patients with diabetes (4,5). A number of potential mechanisms have been suggested to explain the beneficial effects on CV outcomes (8,20–23). One of these is the reduction of plasma volume due to osmotic diuresis, followed by reduced preload, decreased arterial stiffness and blood pressure, which, in turn, would reduce afterload, leading to an improved coronary circulation with increased subendocardial blood flow (20). Increased hematocrit and an increase in plasma levels of ketones have been suggested to improve oxygenation and mitochondrial energy metabolism of the myocardium, respectively (8,14,21,24). A recent meta-analysis has demonstrated a possible renoprotective effect (6,23). However, the CV and renoprotective mechanisms are still unclear and go beyond effects on glucose control, and, based on the fast onset, primary effects on atherosclerosis are unlikely. Therefore, this study focuses on the early effects, within weeks, of SGLT2 inhibition on cardiac function and metabolism. An additional hypothesis is based on the increased urinary glucose excretion caused by SGLT2 inhibition, which leads to enhanced night-time catabolism, followed by increased glycogenolysis, gluconeogenesis, and production of ketone bodies (24). The increased gluconeogenesis during night-time would inhibit mammalian target of rapamycin (mTOR), which would increase autophagy/mitophagy of damaged mitochondria and biogenesis, as well as fusion of mitochondria, maximizing bioenergetic efficiency (22,25), and, possibly, improved myocardial efficiency and contractile work, which will be investigated in this study.

In the current DAPACARD study, we explore in depth the effect of the SGLT2 inhibitor dapagliflozin on myocardial contractile work, myocardial efficiency, substrate uptake, and cardiac metabolism with MRI, PET, and biomarkers and may help unravel these, and other, effects of dapagliflozin.

In conclusion, the overall aim with the DAPACARD study is to gain further knowledge about beneficial effects of dapagliflozin on CV outcomes by advanced imaging with MRI and PET as well as biomarkers. This will potentially help laying the foundation to tailor treatment strategies with SGLT2 inhibitors in patients with T2DM in the future.

## References

[1] Haffner SM, Lehto S, Rönnemaa T, Pyörälä K, Laakso M Mortality from coronary heart disease in subjects with type 2 diabetes and in nondiabetic subjects with and without prior myocardial infarction. N Engl J Med. 1998;339:229–34. 9673301 10.1056/NEJM199807233390404

[2] Effect of intensive blood-glucose control with metformin on complications in overweight patients with type 2 diabetes (UKPDS 34). Lancet. 1998;352:854–65. 9742977

[3] Marso SP, Daniels GH, Brown-Frandsen K, Kristensen P, Mann JFE, Nauck MA, et al. Liraglutide and cardiovascular outcomes in type 2 diabetes. N Engl J Med. 2016;375:311–22. 27295427 10.1056/NEJMoa1603827 PMC4985288

[4] Zinman B, Wanner C, Lachin JM, Fitchett D, Bluhmki E, Hantel S, et al. Empagliflozin, cardiovascular outcomes, and mortality in type 2 diabetes. N Engl J Med. 2015;373:2117–28. 26378978 10.1056/NEJMoa1504720

[5] Neal B, Perkovic V, Mahaffey KW, de Zeeuw D, Fulcher G, Erondu N, et al. Canagliflozin and cardiovascular and renal events in type 2 diabetes. N Engl J Med. 2017;377:644–57. 28605608 10.1056/NEJMoa1611925

[6] Rådholm K, Figtree G, Perkovic V, Solomon SD, Mahaffey KW, de Zeeuw D, et al. Canagliflozin and heart failure in type 2 diabetes mellitus: results from the CANVAS program (Canagliflozin Cardiovascular Assessment Study). 2018; Circulation. Mar 11 [Epub ahead of print]. 10.1161/CIRCULATIONAHA.118.034222 PMC6075881 29526832

[7] Raz I, Mosenzon O, Bonaca MP, Cahn A, Kato ET, Silverman MG, et al. DECLARE-TIMI 58: participants’ baseline characteristics. Diabetes Obes Metab. 2018;20:1102–10. 29322605 10.1111/dom.13217

[8] Inzucchi SE, Zinman B, Fitchett D, Wanner C, Ferrannini E, Schumacher M, et al. How does empagliflozin reduce cardiovascular mortality? Insights from a mediation analysis of the EMPA-REG OUTCOME Trial. Dia Care. 2018;41:356–63. 10.2337/dc17-1096 29203583

[9] Kalam K, Otahal P, Marwick TH Prognostic implications of global LV dysfunction: a systematic review and meta-analysis of global longitudinal strain and ejection fraction. Heart. 2014;100:1673–80. 24860005 10.1136/heartjnl-2014-305538

[10] Mather KJ, Hutchins GD, Perry K, Territo W, Chisholm R, Acton A, et al. Assessment of myocardial metabolic flexibility and work efficiency in human type 2 diabetes using 16-[18 F]fluoro-4-thiapalmitate, a novel PET fatty acid tracer. Am J Physiol Metab. 2016;310:E452–60. 10.1152/ajpendo.00437.2015 PMC4796267 26732686

[11] Hansson NH, Tolbod L, Harms J, Wiggers H, Kim WY, Hansen E, et al. Evaluation of ECG-gated [11C]acetate PET for measuring left ventricular volumes, mass, and myocardial external efficiency. J Nucl Cardiol. 2016;23:670–9. 27094041 10.1007/s12350-015-0331-0

[12] Levelt E, Rodgers CT, Clarke WT, Mahmod M, Ariga R, Francis JM, et al. Cardiac energetics, oxygenation, and perfusion during increased workload in patients with type 2 diabetes mellitus. Eur Heart J. 2016;37:3461–9. 26392437 10.1093/eurheartj/ehv442 PMC5201143

[13] Leung M, Wong VW, Hudson M, Leung DY Impact of improved glycemic control on cardiac function in type 2 diabetes mellitus. Circ Cardiovasc Imaging. 2016;9:e003643. 26962125 10.1161/CIRCIMAGING.115.003643

[14] Neubauer S The failing heart-an engine out of fuel. N Engl J Med. 2007;356:1140–51. 17360992 10.1056/NEJMra063052

[15] Tuunanen H, Engblom E, Naum A, Någren K, Hesse B, Airaksinen KEJ, et al. Free fatty acid depletion acutely decreases cardiac work and efficiency in cardiomyopathic heart failure. Circulation. 2006;114:2130–7. 17088453 10.1161/CIRCULATIONAHA.106.645184

[16] Singh A, Steadman CD, Khan JN, Horsfield MA, Bekele S, Nazir SA, et al. Intertechnique agreement and interstudy reproducibility of strain and diastolic strain rate at 1.5 and 3 tesla: a comparison of feature-tracking and tagging in patients with aortic stenosis. J Magn Reson Imaging. 2015;41:1129–37. 24700404 10.1002/jmri.24625

[17] Gjesdal O, Yoneyama K, Mewton N, Wu C, Gomes AS, Hundley G, et al. Reduced long axis strain is associated with heart failure and cardiovascular events in the multi-ethnic study of Atherosclerosis. J Magn Reson Imaging. 2016;44:178–85. 26731196 10.1002/jmri.25135

[18] Tuunanen H, Kuusisto J, Toikka J, Jääskeläinen P, Marjamäki P, Peuhkurinen K, et al. Myocardial perfusion, oxidative metabolism, and free fatty acid uptake in patients with hypertrophic cardiomyopathy attributable to the Asp175Asn mutation in the alpha-tropomyosin gene: a positron emission tomography study. J Nucl Cardiol. 2007;14:354–65. 17556170 10.1016/j.nuclcard.2006.12.329

[19] Viljanen APM, Karmi A, Borra R, Pärkkä JP, Lepomäki V, Parkkola R, et al. Effect of caloric restriction on myocardial fatty acid uptake, left ventricular mass, and cardiac work in obese adults. Am J Cardiol. 2009;103:1721–6. 19539082 10.1016/j.amjcard.2009.02.025

[20] Lytvyn Y, Bjornstad P, Udell JA, Lovshin JA, Cherney DZI Sodium glucose cotransporter-2 inhibition in heart failure: potential mechanisms, clinical applications, and summary of clinical trials. Circulation. 2017;136:1643–58. 29061576 10.1161/CIRCULATIONAHA.117.030012 PMC5846470

[21] Ferrannini E Sodium-glucose co-transporters and their inhibition: clinical physiology. Cell Metab. 2017;26:27–38. 28506519 10.1016/j.cmet.2017.04.011

[22] Esterline RL, Vaag A, Oscarsson J, Vora J Mechanisms in endocrinology: SGLT2 inhibitors: clinical benefits by restoration of normal diurnal metabolism? Eur J Endocrinol. 2018;178:R113–25. 29371333 10.1530/EJE-17-0832

[23] Jardine MJ, Mahaffey KW, Neal B, Agarwal R, Bakris GL, Brenner BM, et al. The Canagliflozin and Renal Endpoints in Diabetes with Established Nephropathy Clinical Evaluation (CREDENCE) study rationale, design, and baseline characteristics. Am J Nephrol. 2017;46:462–72. 29253846 10.1159/000484633 PMC5804835

[24] Ferrannini E, Muscelli E, Frascerra S, Baldi S, Mari A, Heise T, et al. Metabolic response to sodium-glucose cotransporter 2 inhibition in type 2 diabetic patients. J Clin Invest. 2014;124:499–508. 24463454 10.1172/JCI72227 PMC3904627

[25] Rambold AS, Cohen S, Lippincott-Schwartz J Fatty acid trafficking in starved cells: regulation by lipid droplet lipolysis, autophagy, and mitochondrial fusion dynamics. Dev Cell. 2015;32:678–92. 25752962 10.1016/j.devcel.2015.01.029 PMC4375018

